# Association of Baseline and Serial High-Sensitivity Cardiac Troponin T with In-Hospital Mortality Across Clinical Presentation, LVEF and Diabetes Status in Decompensated Heart Failure

**DOI:** 10.3390/biomedicines14102369

**Published:** 2026-10-17

**Authors:** Mara Diaconu, Dan-Cristian Popescu, Diana Țînț, Alexandru-Cristian Nechita

**Affiliations:** 1 Department of Cardiology, Clinical Emergency Hospital Sfântul Pantelimon, 021659 Bucharest, Romania; mara2228@gmail.com (M.D.); popescu_dan_95@yahoo.com (D.-C.P.); cardionechita@yahoo.com (A.-C.N.); 2 Faculty of Medicine, Carol Davila University of Medicine and Pharmacy, 020021 Bucharest, Romania; 3 Faculty of Medicine, Transilvania University, 500036 Brașov, Romania; 4 Department of Cardiology, ICCO Clinics, 500059 Brașov, Romania

**Keywords:** decompensated heart failure, high-sensitivity cardiac troponin T, in-hospital mortality, myocardial injury, clinical presentation, left ventricular ejection fraction, diabetes status, risk stratification

## Abstract

**Background/Objectives**: High-sensitivity cardiac troponin T (hs-cTnT) is frequently elevated in decompensated heart failure (DHF), but its prognostic association may vary according to clinical context. We evaluated baseline and serial hs-cTnT in relation to in-hospital mortality across clinical presentation, left ventricular ejection fraction (LVEF) and diabetes status. **Methods**: A total of 653 patients hospitalized with DHF were prospectively analyzed. Baseline hs-cTnT was assessed globally and across clinical presentation, LVEF and diabetes status. Among 334 patients with repeated measurements, serial hs-cTnT was evaluated using continuous measures and threshold-based trajectories. **Results**: In-hospital mortality was 9.0%. Baseline hs-cTnT showed moderate discrimination for mortality (AUC 0.658). The internally derived 38.4 pg/mL threshold identified higher mortality above versus below the threshold (14.8% vs. 5.1%, *p* < 0.001) but showed limited bootstrap stability. Clinical presentation significantly modified the association between hs-cTnT and mortality (global interaction *p* = 0.022), driven by a stronger association in decompensated right-sided versus left-sided HF (ratio of ORs 2.77, 95% CI 1.26–6.07). No significant modification effect was observed by LVEF or diabetes status. In a serial subgroup, repeated hs-cTnT remained associated with mortality after multivariable adjustment (OR 1.59 per doubling, 95% CI 1.14–2.21), although the increase in discrimination over the baseline model was not significant (AUC 0.750 vs. 0.777, *p* = 0.205). The above-above trajectory remained associated with mortality in Firth regression (adjusted OR 2.32, 95% CI 1.02–5.61). **Conclusions**: hs-cTnT was associated with in-hospital mortality in DHF, with heterogeneity by clinical presentation. Serial measurements were associated with outcome, but their incremental discrimination value was limited. Threshold-based trajectories should be considered exploratory pending external validation.

## 1. Introduction

Decompensated heart failure (DHF) is a clinically heterogeneous syndrome characterized by acute or progressive worsening of symptoms and/or signs of heart failure (HF) requiring urgent medical assessment and treatment intensification [1]. Patients hospitalized with HF decompensation exhibit substantial heterogeneity in clinical presentation, underlying cardiac substrate, comorbidity burden and short-term outcome [2,3]. Early prognostic stratification remains a major priority and multiple clinical variables, biomarkers or risk scores have been evaluated to improve prognostic assessment in patients hospitalized with DHF [4,5]. 

Clinical presentation at admission represents a practical and clinically relevant framework for characterizing the heterogeneity of DHF. According to the 2026 ESC Guidelines, four principal clinical presentations are recognized: decompensated left-sided HF, decompensated right-sided HF, acute pulmonary edema (APE) and cardiogenic shock [1]. These presentations differ in their predominant pathophysiological mechanisms, congestion and perfusion profiles, clinical severity, and therapeutic priorities, although overlap between categories may occur [1]. Studies based on phenotyping patients in the emergency department (ED) have shown that clinical profiles defined at presentation are associated with different management patterns and outcomes [6,7]. 

Echocardiography has a central role in the evaluation of patients with DHF, as it provides rapid, non-invasive assessment of cardiac structure, ventricular function, filling pressures and right-heart involvement [8,9]. Among echocardiographic measurements, left ventricular ejection fraction (LVEF) remains a central parameter for HF classification, distinguishing HF with reduced ejection fraction (HFrEF), characterized by LVEF < 50% and HF with preserved ejection fraction (HFpEF), characterized by LVEF ≥ 50% [1]. Recent data suggest that the prognostic impact of circulating biomarkers may vary across LVEF spectrum [10].

Non-cardiac comorbidity burden represents an important contributor to DHF heterogeneity [11]. Diabetes mellitus is common among patients hospitalized with DHF and has been associated with adverse in-hospital and post-discharge outcomes [12,13]. Its prognostic impact may vary across LVEF spectrum, with prior data suggesting phenotype-specific differences in its association with outcomes [14]. In HFpEF, diabetes is increasingly recognized as part of a broader cardiometabolic substrate characterized by metabolic dysfunction, microvascular impairment, systemic inflammation and myocardial remodeling [15,16,17].

Within this heterogeneous framework, high-sensitivity cardiac troponin T (hs-cTnT) has emerged as an important biomarker of myocardial injury in DHF, with adverse outcomes, including increased in-hospital mortality [18]. Troponin elevation is frequently observed in patients with DHF and may reflect several mechanisms, including increased wall stress, neurohormonal activation, subendocardial ischemia, inflammation, oxygen supply-demand mismatch or renal dysfunction [19,20]. The use of high-sensitivity assays has expanded the ability to detect low-grade myocardial injury, and both baseline and serial hs-cTnT measurements during hospitalization have been shown to provide prognostic value in patients with DHF [21,22,23].

Despite the established association of hs-cTnT with adverse outcomes in DHF, it remains uncertain whether this association differs across contemporary clinical presentation and LVEF categories or according to diabetes status. Therefore, we evaluated the association of baseline and serial hs-cTnT with in-hospital mortality and formally tested effect modification by clinical presentation, LVEF category and diabetes status.

## 2. Materials and Methods

### 2.1. Study Population

This prospective, single-center observational study included 653 patients hospitalized with DHF in the Cardiology Department of the Clinical Emergency Hospital “Sf. Pantelimon” Bucharest between January 2025 and May 2026. HF decompensation was established through an integrated assessment of presenting signs and symptoms, natriuretic peptide concentrations, and echocardiographic evidence of cardiac dysfunction. Clinical presentation and LVEF categories were defined according to the 2026 ESC Guidelines for the management of heart failure [1]. All-cause mortality during the index hospitalization was defined as the primary outcome. 

The inclusion criteria required complete clinical, laboratory and echocardiographic assessment, together with an available baseline hs-cTnT measurement at admission. Patients with incomplete data, as well as patients with an acute coronary syndrome (ACS) diagnosis during hospitalization were excluded from the study population. ACS was adjudicated by the treating cardiology team based on an integrated assessment of ischemic symptoms, serial electrocardiographic findings, hs-cTnT kinetics and echocardiographic evidence of new regional wall-motion abnormalities. Patients were excluded when an elevated hs-cTnT concentration with significant rise and/or fall was accompanied by clinical, electrocardiographic or echocardiographic evidence of acute myocardial ischemia. An elevated hs-cTnT concentration or a dynamic change in hs-cTnT alone, in the absence of clinical, electrocardiographic or echocardiographic evidence of acute myocardial ischemia, was considered myocardial injury rather than ACS and did not constitute an exclusion criterion. Coronary imaging or angiography was incorporated into the diagnostic assessment when clinically indicated and available. Acute myocardial injury related to hemodynamic stress, tachyarrhythmia, hypoxemia, renal dysfunction or other conditions accompanying decompensated HF was retained when no evidence of an ACS was present. Cases fulfilling criteria for myocardial infarction, including type 2 myocardial infarction related to oxygen supply-demand imbalance when accompanied by evidence of acute myocardial ischemia, were excluded, whereas acute myocardial injury without evidence of ischemia remained eligible [24].

For serial hs-cTnT analysis, only patients with a second measurement of hs-cTnT during hospitalization were included. Those without repeated measurements were excluded from this analysis but remained eligible for the baseline hs-cTnT evaluation.

This study protocol complied with the ethical principles of the Declaration of Helsinki and received approval from the Ethics Committee of Clinical Emergency Hospital “Sf. Pantelimon” Bucharest.

### 2.2. Data Collection 

At hospital presentation, patients underwent a standardized clinical evaluation by a cardiologist. Demographic information, cardiovascular risk factors, relevant medical history, and vital signs were recorded at admission. Collected risk factors included hypertension, dyslipidemia, diabetes mellitus, obesity and smoking, while documented comorbidities included previous HF, atrial fibrillation, ischemic heart disease, chronic kidney disease and chronic obstructive pulmonary disease. Clinical data included heart rate, systolic blood pressure (SBP), diastolic blood pressure, presence of jugular venous distension and peripheral edema. The laboratory dataset comprised hemoglobin, serum sodium, serum creatinine, urea, N-terminal pro-B-type Natriuretic Peptide (NT-proBNP) and hs-cTnT. HF etiology was additionally categorized as ischemic or non-ischemic according to the documented underlying etiology in the medical record.

Transthoracic echocardiography was performed during the initial ED assessment using a GE Logiq S7 XDclear 2.0 ultrasound system (GE Healthcare, Wauwatosa, WI, USA). Examinations were performed and interpreted by cardiologists experienced in transthoracic echocardiography, before the subsequent in-hospital outcome was known. LVEF was quantified using the biplane Simpson method from apical four- and two-chamber views. Recorded echocardiographic parameters included indices of left and right ventricular systolic and diastolic function, left ventricular filling pressures, tricuspid annular plane systolic excursion (TAPSE), right-ventricular-right atrial (RV-RA) pressure gradient and inferior vena cava (IVC) diameter and the presence and severity of tricuspid regurgitation. TAPSE was measured by M-mode at the lateral tricuspid annulus. The RV-RA pressure gradient was estimated from peak tricuspid regurgitation velocity using the simplified Bernoulli equation, while IVC diameter was assessed from the subcostal view during spontaneous respiration [25,26]. Baseline hs-cTnT sampling and transthoracic echocardiography were both performed at presentation in the ED as part of the initial clinical assessment.

### 2.3. Clinical Presentation and LVEF Classification

Patients were classified according to three clinically relevant axes: clinical presentation, LVEF category and diabetes status.

Clinical presentation was defined according to the form of presentation:

- Decompensated left-sided HF
- Decompensated right-sided HF
- APE
- Cardiogenic shock

DHF was defined as worsening signs or symptoms of HF requiring hospitalization. To obtain mutually exclusive analytical categories, patients presenting with APE or cardiogenic shock were assigned to these groups first. APE was defined as acute respiratory distress associated with clinical and/or radiological evidence of pulmonary congestion requiring urgent therapy. Cardiogenic shock was defined as hypotension associated with clinical signs of hypoperfusion requiring urgent hemodynamic support. Predominantly right-sided HF was operationally defined by the concomitant presence of right ventricular systolic dysfunction (TAPSE < 17 mm), jugular vein distension and at least one additional marker of right-sided pressure or volume overload/systemic venous congestion: an IVC diameter > 21 mm or tricuspid regurgitation [27]. Patients not fulfilling these criteria were classified as decompensated left-sided HF.

LVEF was defined according to guideline-based categories:

- HFrEF: LVEF < 50%
- HFpEF: LVEF ≥ 50%

Diabetes status was defined by a previously documented diagnosis of diabetes mellitus.

### 2.4. hs-cTnT Assessment

Hs-cTnT concentrations were measured using the Elecsys Troponin T electrochemiluminescence immunoassay (Roche Diagnostics, Mannheim, Germany) on a cobas e immunoassay platform. Results were expressed in pg/mL. According to the manufacturer, the assay has a 99th-percentile upper reference limit (URL) of 14 pg/mL, a limit of blank of 3 pg/mL, a limit of detection of 5 pg/mL and a limit of quantification of 13 pg/mL at a coefficient of variation ≤10%, with a measuring range of 3–10,000 pg/mL [28,29]. 

Baseline hs-cTnT was defined as the value obtained at presentation in the ED. In patients with repeated hs-cTnT measurements, repeat sampling was not mandated by a predefined study protocol but was performed at the discretion of the treating physician as part of routine clinical care. The median documented time to repeat measurement was 13.5 h (IQR 7–22 h; range 5–56 h). Peak hs-cTnT value was defined as the highest value recorded between baseline and repeated hs-cTnT measurements. Absolute hs-cTnT change was calculated as the absolute difference between repeated and baseline hs-cTnT values, whereas relative hs-cTnT change was expressed as percentage change from baseline.

### 2.5. Statistical Analysis

Continuous variables were expressed as mean ± standard deviation (SD) or median with interquartile range (IQR), depending on their distribution, while categorical variables were presented as numbers and percentages. Differences between survivors and non-survivors were assessed using the independent-samples *t* test or Mann–Whitney U test for continuous variables and the chi-square test or Fisher’s exact test for categorical variables, as appropriate. A two-sided *p*-value < 0.05 was considered statistically significant. 

Receiver operating characteristic (ROC) curve analysis was performed to assess the ability of baseline hs-cTnT to discriminate in-hospital mortality. The area under the curve (AUC) was calculated with 95% confidence intervals (CI), and the optimal baseline hs-cTnT cut-off was determined using the Youden index. The resulting threshold was considered an internally derived exploratory prognostic threshold and was distinguished from the assay-specific 99th-percentile URL. To reduce reliance on dichotomization, baseline hs-cTnT was additionally analyzed as a log2-transformed continuous variable, with the corresponding odds ratio (OR) representing the change in mortality odds associated with each doubling of hs-cTnT concentration. Sensitivity analyses were also performed using the assay-specific 99th-percentile URL of 14 pg/mL. The stability of the internally derived prognostic threshold was assessed using non-parametric bootstrapping with 2000 resamples, with the optimal Youden threshold recalculated in each bootstrap sample. 

The relationship between baseline hs-cTnT and NT-proBNP was explored using Spearman correlation. To assess whether baseline hs-cTnT provided prognostic information beyond NT-proBNP, a logistic regression model including log2-transformed NT-proBNP was compared with an extended model additionally including log2-transformed baseline hs-cTnT. Nested models were compared using the likelihood-ratio test, while changes in discrimination were evaluated by ROC AUC comparison using the DeLong method. Overall predictive accuracy was additionally assessed using the Brier score.

Mortality according to hs-cTnT category was compared using the chi-square or Fisher’s exact test, as appropriate, and associations were expressed as ORs with 95% CI. Subgroup analyses were performed according to clinical presentation, LVEF category and diabetes status. Within the HFpEF subgroup, an exploratory analysis further stratified patients according to LVEF 50–64% and supra-normal LVEF ≥ 65%. Effect modification by supra-normal LVEF status was assessed using an interaction term between log2-transformed baseline hs-cTnT and LVEF subgroup. 

Effect modification was formally assessed by introducing interaction terms between baseline hs-cTnT and each stratification axis in logistic regression models. Baseline hs-cTnT was log2-transformed because of its right-skewed distribution, allowing the corresponding effect estimate to be interpreted per doubling of hs-cTnT concentration. For clinical presentation, the overall interaction was assessed using a likelihood-ratio test comparing models with and without the hs-cTnT-by-presentation interaction terms. Interaction terms were similarly evaluated for LVEF category and diabetes status. As a sensitivity analysis, interaction testing was repeated using the internally derived hs-cTnT threshold of 38.4 pg/mL. Differences in the discriminatory performance of baseline hs-cTnT across stratification groups were formally assessed by comparing the corresponding ROC AUCs. For clinical presentation, an overall comparison across the four presentation categories was performed. Because the stratification groups were mutually exclusive, differences between AUCs were assessed using independent-sample non-parametric U-statistic variance estimates for independent groups. An omnibus Wald chi-square test was used to assess equality of AUCs across the four clinical presentation categories, followed by pairwise comparisons using decompensated left-sided HF as the reference category. AUCs were also compared between HFrEF and HFpEF and between patients with and without diabetes.

All subgroup analyses were considered exploratory. Given the multiple subgroup comparisons and the limited number of outcome events in some strata, emphasis was placed on effect estimates with 95% CI and formal interaction testing rather than on isolated subgroup-specific *p*-values. No formal adjustment for multiple comparisons was applied, and subgroup-specific *p*-values were therefore interpreted cautiously. 

In patients with repeated hs-cTnT measurements, ROC curve analysis was performed for baseline, repeated and peak hs-cTnT values, as well as for absolute and relative changes in hs-cTnT. Serial hs-cTnT patterns were classified as below-below, below-above, above-above and above-below according to whether values were below or above the identified threshold at baseline and at the repeated measurement. Below-below indicated values below the threshold at both time points; below-above indicated a value below the threshold at baseline and above it at the repeated measurement; above-above indicated values above the threshold at both measurements; and above-below indicated a value above the threshold at baseline and below it at the repeated measurement.

Pairwise comparisons between the ROC AUCs of baseline, repeated and peak hs-cTnT were performed using the DeLong test for correlated ROC curves [30]. To assess whether repeated hs-cTnT provided prognostic information beyond baseline assessment, a parsimonious baseline logistic regression model including age, SBP, log2-transformed serum creatinine, log2-transformed NT-proBNP, hemoglobin and log2-transformed baseline hs-cTnT was compared with an extended serial model additionally including log2-transformed repeated hs-cTnT. Nested models were compared using the likelihood-ratio test. Changes in discrimination were assessed by ROC AUC comparison, and overall predictive accuracy was evaluated using the Brier score. Internal validation was performed using 1000 bootstrap resamples to estimate optimism-corrected discrimination and calibration [31]. 

Given the limited number of in-hospital deaths in the subgroup with serial hs-cTnT, a parsimonious bias-reduced Firth logistic regression model was used to reduce small-sample bias and limit overfitting [32]. The above-above hs-cTnT pattern was entered as the exposure of interest. Covariates were selected on clinical grounds for the revised analysis rather than through univariable significance testing and included age, SBP, serum creatinine, NT-proBNP and hemoglobin, representing baseline risk, hemodynamic status, renal function, heart failure severity and systemic disease burden, respectively. Serum creatinine and NT-proBNP were log2-transformed because of their right-skewed distribution. The corresponding ORs therefore represent the effect associated with a doubling of each biomarker. Age and systolic blood pressure (SBP) were modeled per 10-unit increase. Results are reported as adjusted ORs with 95% profile penalized-likelihood CI. Sensitivity analyses evaluating TAPSE, RV-RA gradient and urea were performed using similarly constrained models to assess the robustness of the primary association without excessive model complexity. 

The above-above hs-cTnT pattern was compared with all other serial patterns combined, both in the overall serial-measurement cohort and across clinical presentation, LVEF, and diabetes subgroups.

All statistical analyses were performed using IBM SPSS Statistics for Windows, version 26.0 (IBM Corp., Armonk, NY, USA) and R statistical software, version 4.6.1 (R Foundation for Statistical Computing, Vienna, Austria). 

## 3. Results

### 3.1. Baseline Characteristics

653 patients hospitalized with DHF were analyzed: 59 patients (9.0%) died during hospitalization, whereas 594 patients (91.0%) survived. Baseline characteristics of the study population are summarized in Table 1. Non-survivors were significantly older than survivors (77.7 ± 9.5 vs. 74.0 ± 11.9 years, *p* = 0.007). The most frequent form of presentation was decompensated left-sided HF (66.9%), followed by APE (19.4%), decompensated right-sided HF (11.0%) and cardiogenic shock (2.6%). The distribution of clinical presentations differed significantly according to in-hospital outcome (*p* < 0.001). Ischemic HF etiology was present in 332 patients (50.8%) and was more frequent among non-survivors than survivors (69.5% vs. 49.0%, *p* = 0.003).

Non-survivors had lower SBP (135.7 ± 31.1 vs. 147.8 ± 31.9 mmHg, *p* = 0.006), lower hemoglobin levels (11.3 ± 2.4 vs. 12.6 ± 2.4 g/dL, *p* = 0.002), higher blood urea levels (97.4 ± 53.5 vs. 65.0 ± 36.3 mg/dL, *p* < 0.001), higher NT-proBNP levels (17,136.2 ± 13,619.7 vs. 8715.3 ± 12,024.1 pg/mL, *p* < 0.001) and higher hs-cTnT levels (92.2 ± 126.9 vs. 48.5 ± 60.9 pg/mL, *p* = 0.011).

Echocardiographically, non-survivors had lower TAPSE values (16.6 ± 5.5 vs. 18.5 ± 5.3 mm, *p* = 0.012) and higher RV-RA gradient (36.3 ± 14.4 vs. 31.5 ± 12.8 mmHg, *p* = 0.016). LVEF (40.3 ± 16.6 vs. 44.2 ± 13.9%, *p* = 0.089) and the distribution of HFpEF and HFrEF were also comparable between outcome groups (*p* = 0.338).

### 3.2. Prognostic Performance of Baseline hs-cTnT for In-Hospital Mortality

**Figure 1:**
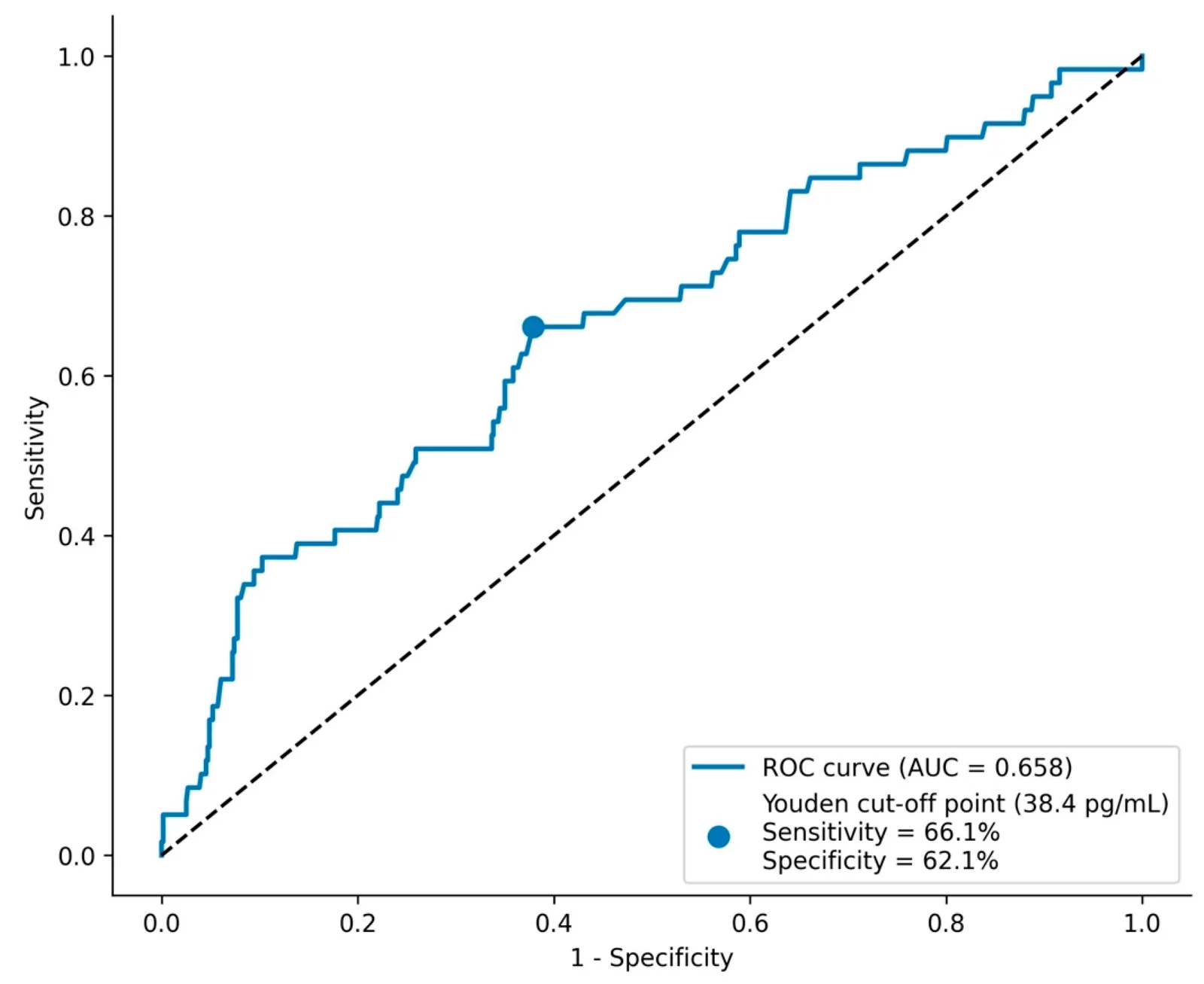
ROC curve of baseline hs-cTnT for in-hospital mortality. The dashed diagonal line represents the line of no discrimination.

In ROC analysis, baseline hs-cTnT showed significant discrimination for in-hospital mortality, with an AUC of 0.658 (Figure 1). The optimal Youden-derived threshold was 38.4 pg/mL, corresponding to a sensitivity of 66.1% and a specificity of 62.1%. 

**Figure 2:**
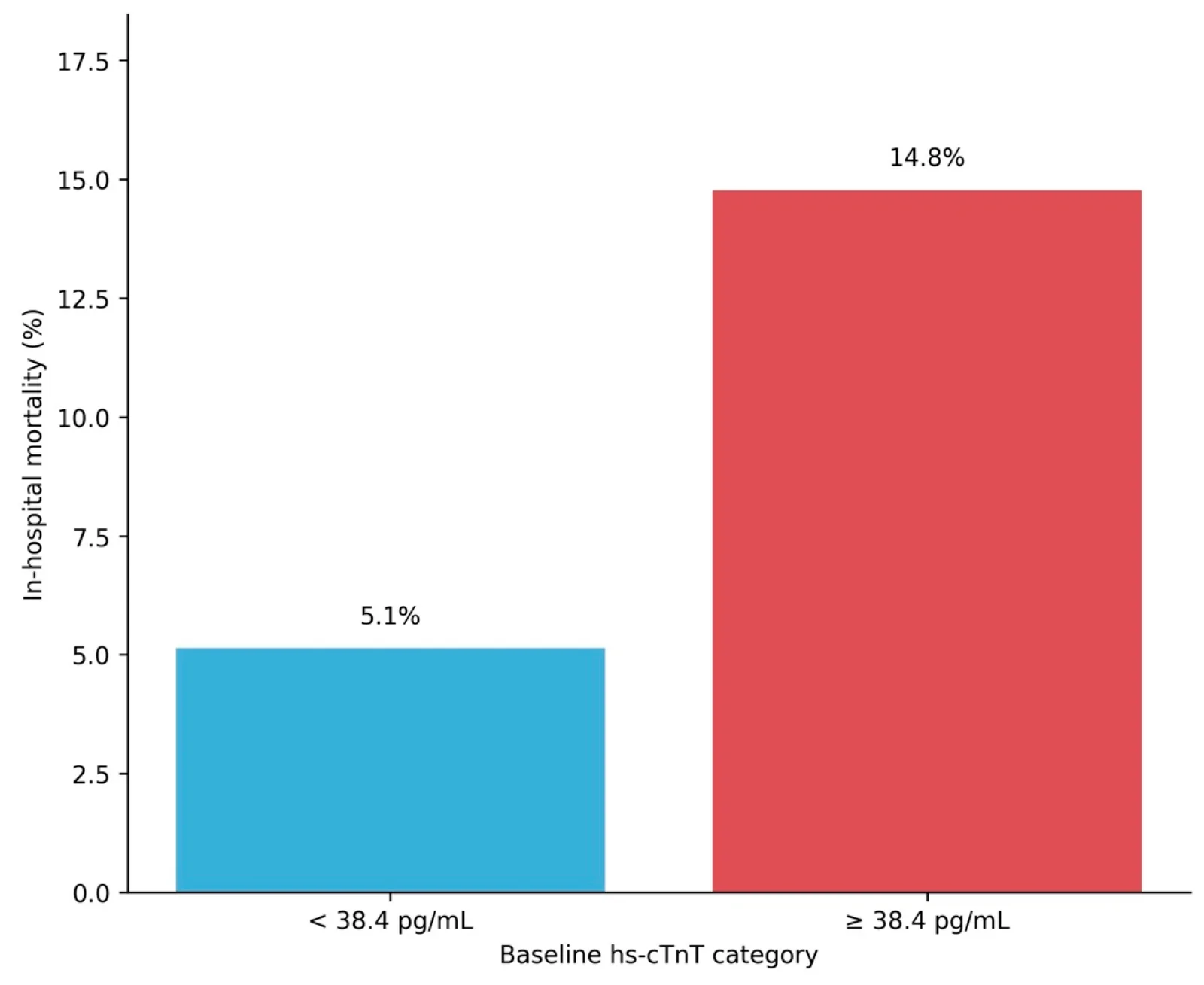
In-hospital mortality by baseline hs-cTnT category.

When stratification was made according to this threshold, patients with baseline hs-cTnT ≥ 38.4 pg/mL had a markedly higher in-hospital mortality rate than those with values below the threshold (14.8% vs. 5.1%; Figure 2). Above-threshold baseline hs-cTnT was associated with an approximately three-fold higher odds of in-hospital mortality (OR 3.20, 95% CI 1.82–5.62, *p* < 0.001).

When baseline hs-cTnT was analyzed as a continuous log2-transformed variable, each doubling in hs-cTnT concentration was associated with higher odds of in-hospital mortality (OR 1.49, 95% CI 1.21–1.84, *p* < 0.001). Using the assay-specific 99th-percentile URL of 14 pg/mL, 573 patients (87.7%) had hs-cTnT concentrations above the URL. In-hospital mortality was 9.4% among patients with hs-cTnT ≥ 14 pg/mL and 6.3% among those with values <14 pg/mL (OR 1.56, 95% CI 0.61–4.03, *p* = 0.357). 

In 2000 bootstrap resamples, the median optimal threshold was 40.3 pg/mL (IQR 38.4–85.6 pg/mL), with a 95% bootstrap percentile interval of 27.0–91.4 pg/mL. The optimism-corrected Youden index was 0.237, compared with an apparent value of 0.282, indicating limited stability of the data-derived threshold and supporting its interpretation as exploratory.

Baseline hs-cTnT and NT-proBNP showed a moderate positive correlation (Spearman ρ = 0.340, *p* < 0.001). When both biomarkers were entered as log2-transformed continuous variables in the same logistic regression model, NT-proBNP remained significantly associated with in-hospital mortality (OR 1.66 per doubling, 95% CI 1.36–2.03, *p* < 0.001), whereas the association with baseline hs-cTnT was attenuated (OR 1.24 per doubling, 95% CI 0.99–1.56, *p* = 0.063). Addition of baseline hs-cTnT to the NT-proBNP-only model did not significantly improve overall model fit (likelihood-ratio χ^2^ = 3.48, *p* = 0.062). The AUC increased marginally from 0.746 (95% CI 0.682–0.811) to 0.749 (95% CI 0.683–0.815), with no significant improvement in discrimination (ΔAUC 0.003, DeLong *p* = 0.846). The Brier score decreased slightly from 0.0775 to 0.0760. 

### 3.3. Prognostic Performance of Baseline hs-cTnT Across Clinical Presentation, LVEF Category and Diabetes Status

#### 3.3.1. Prognostic Performance According to Clinical Presentation 

Baseline hs-cTnT was evaluated separately across the four clinical presentation categories (Table 2). In patients with predominantly decompensated right-sided HF, baseline hs-cTnT showed significant discrimination for in-hospital mortality, with an AUC of 0.841 (95% CI 0.705–0.976, *p* < 0.001). In decompensated left-sided HF, the AUC was 0.603 (95% CI 0.488–0.717, *p* = 0.069). Among patients presenting with APE, AUC was 0.669 (95% CI 0.427–0.912, *p* = 0.164), whereas in cardiogenic shock the estimate was imprecise and did not demonstrate significant discrimination in the small subgroup (AUC 0.485, 95% CI 0.189–0.781, *p* = 0.960). Event counts were limited in the APE and cardiogenic shock subgroups, with 6 and 11 in-hospital deaths, respectively, resulting in wide CI for subgroup-specific estimates.

**Figure 3:**
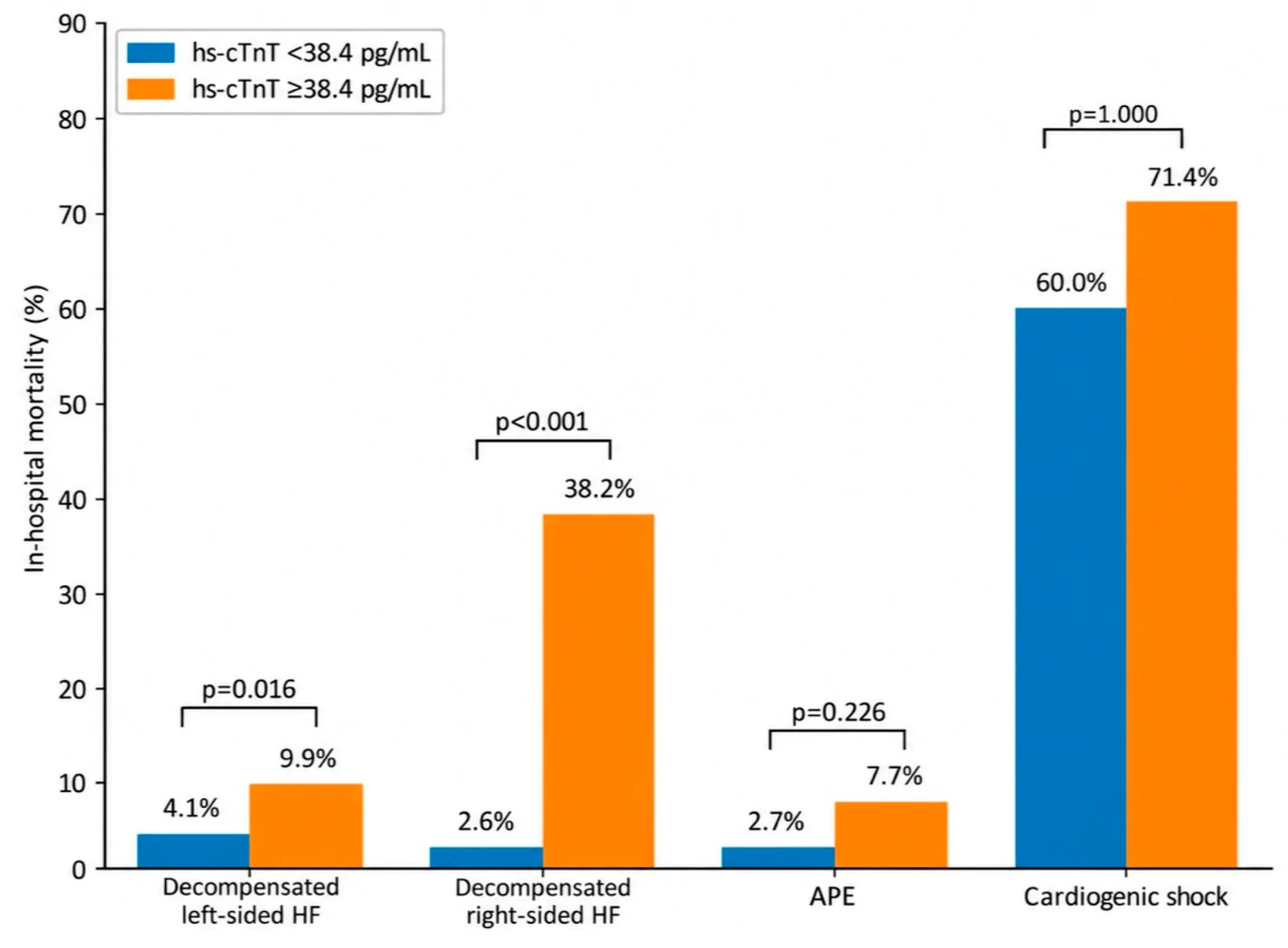
In-hospital mortality according to baseline hs-cTnT cut-off and clinical presentation.

Using the baseline hs-cTnT cut-off of 38.4 pg/mL, in-hospital mortality was higher among patients with above-threshold hs-cTnT in decompensated left-sided HF (9.9% vs. 4.1%, *p* = 0.016) and markedly higher in predominantly decompensated right-sided HF (38.2% vs. 2.6%, *p* < 0.001). In patients with APE, mortality was numerically higher above the hs-cTnT threshold (7.7% vs. 2.7%), although the difference was not statistically significant (*p* = 0.226). No statistically significant difference in mortality according to the study-derived threshold was observed in the cardiogenic shock subgroup (*p* = 1.000) (Figure 3).

#### 3.3.2. Prognostic Performance According to LVEF Category

When evaluated according to LVEF category, baseline hs-cTnT showed significant discrimination for in-hospital mortality in both HFrEF and HFpEF (Table 3). In patients with HFrEF, the AUC was 0.647 (95% CI 0.547–0.747, *p* = 0.003), while in patients with HFpEF, the AUC was 0.669 (95% CI 0.541–0.797, *p* = 0.008).

**Figure 4:**
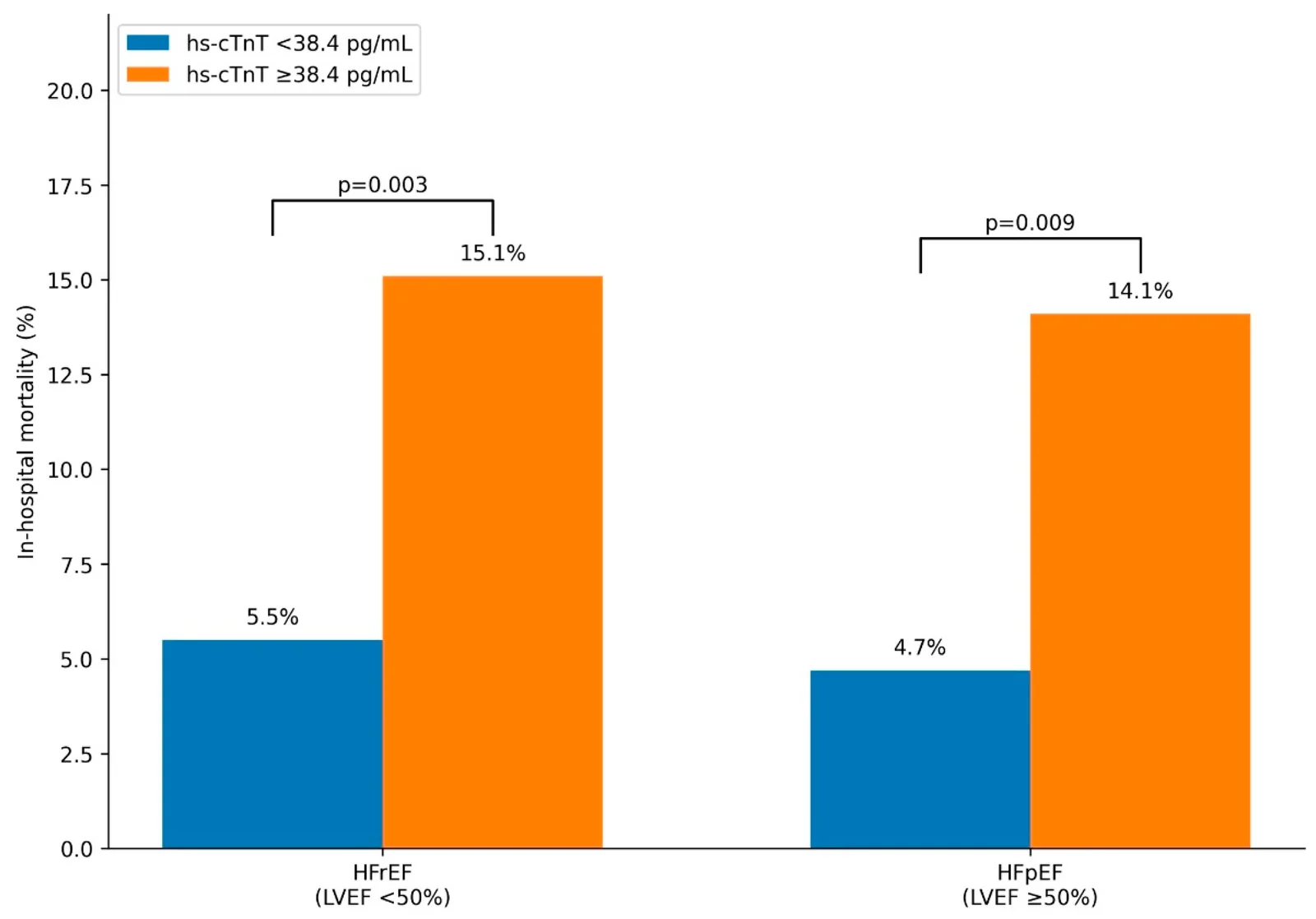
In-hospital mortality according to baseline hs-cTnT cut-off and LVEF category.

Using the baseline hs-cTnT threshold of 38.4 pg/mL, in-hospital mortality was higher among patients with above-threshold hs-cTnT in both LVEF categories. In HFrEF, mortality increased from 5.5% below the threshold to 15.1% above the threshold (*p* = 0.003), while in HFpEF it increased from 4.7% to 14.1%, respectively (*p* = 0.009) (Figure 4).

As an exploratory analysis within the HFpEF population, patients were further stratified according to conventional preserved LVEF (50–64%; *n* = 235) and supra-normal LVEF (≥65%; *n* = 47). In-hospital mortality was 8.1% and 6.4%, respectively. Baseline hs-cTnT showed significant discrimination for in-hospital mortality in the LVEF 50–64% subgroup (AUC 0.689, 95% CI 0.553–0.826, *p* = 0.007), whereas the estimate was imprecise in patients with LVEF ≥ 65% (AUC 0.500, 95% CI 0.159–0.841, *p* = 1.000), among whom only three deaths occurred. No significant interaction was observed between log2-transformed baseline hs-cTnT and supra-normal LVEF status (*p* for interaction = 0.410).

#### 3.3.3. Prognostic Performance According to Diabetes Status 

The prognostic value of baseline hs-cTnT was further evaluated according to diabetes status (Table 4). Significant discrimination for in-hospital mortality was observed both in patients without diabetes (AUC 0.629, 95% CI 0.504–0.739, *p* = 0.023), and in patients with diabetes (AUC 0.679, 95% CI 0.576–0.784, *p* = 0.001). 

**Figure 5:**
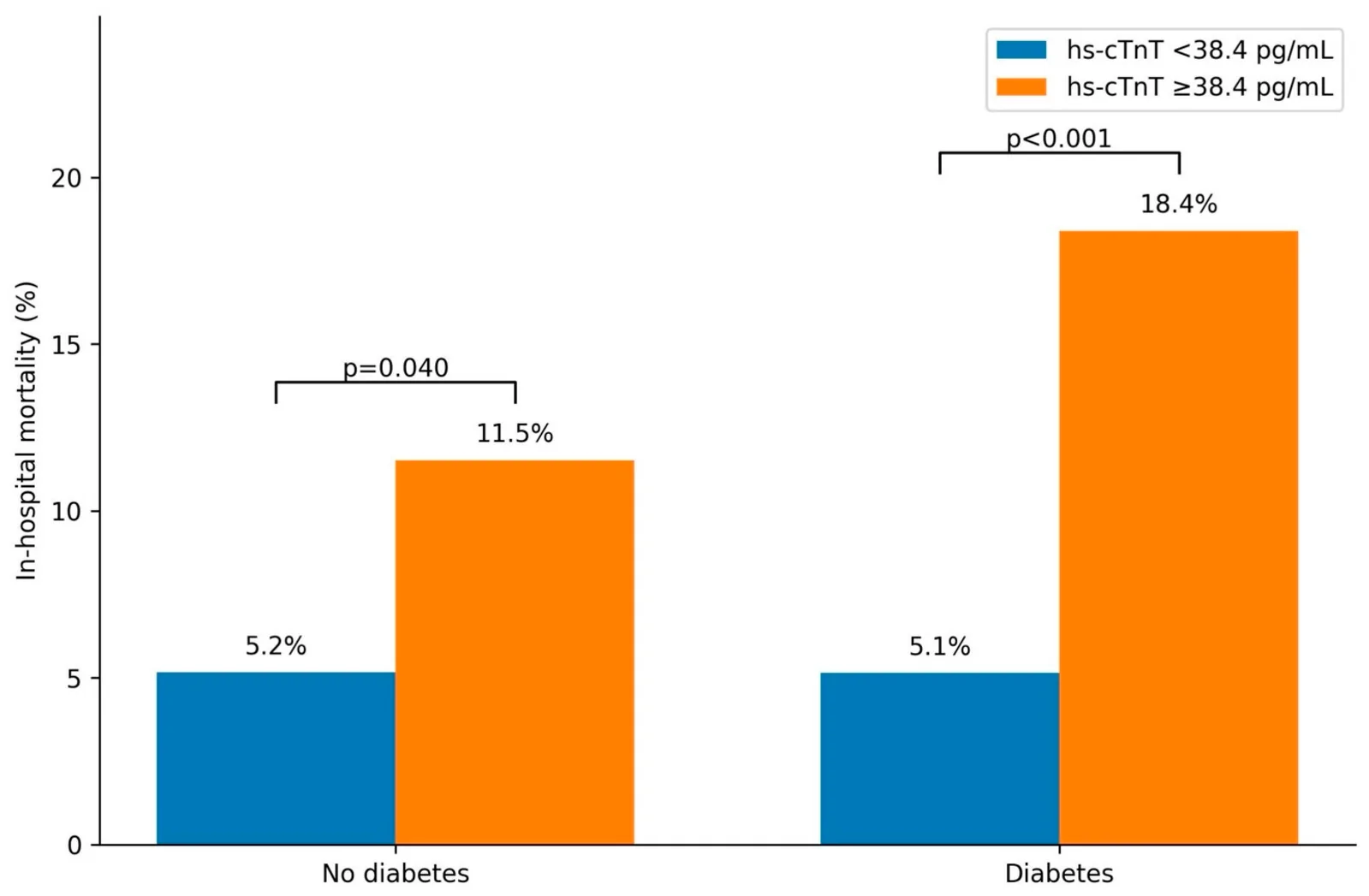
In-hospital mortality according to baseline hs-cTnT cut-off and diabetes status.

Using the baseline hs-cTnT threshold of 38.4 pg/mL, above-threshold hs-cTnT was associated with higher in-hospital mortality in both diabetes-status groups. Among patients without diabetes, mortality was 11.5% above the threshold compared with 5.2% below it (OR 2.40, 95% CI 1.10–5.23, *p* = 0.040). Among patients with diabetes, mortality was 18.4% versus 5.1%, respectively (OR 4.17, 95% CI 1.80–9.69, *p* < 0.001) (Figure 5). 

#### 3.3.4. Interaction Analyses 

Formal interaction testing using log2-transformed baseline hs-cTnT demonstrated a significant interaction between hs-cTnT and clinical presentation (global *p* for interaction = 0.022). Compared with decompensated left-sided HF, the association between hs-cTnT and in-hospital mortality was stronger in decompensated right-sided HF (ratio of ORs 2.77, 95% CI 1.26–6.07, *p* = 0.011), whereas no significant interaction was observed for APE (ratio of ORs 1.35, 95% CI 0.65–2.80, *p* = 0.422) or cardiogenic shock (ratio of ORs 0.81, 95% CI 0.42–1.56, *p* = 0.527). 

No significant effect modification was observed according to LVEF category (*p* for interaction = 0.714) or diabetes status (*p* for interaction = 0.208). In sensitivity analyses using the internally-derived hs-cTnT threshold of 38.4 pg/mL, no statistically significant interactions were observed for clinical presentation (global *p* for interaction = 0.119), LVEF category (*p* for interaction = 0.887) or diabetes status (*p* for interaction = 0.341). 

Formal comparison of discriminatory performance demonstrated significant overall heterogeneity in baseline hs-cTnT AUCs across clinical presentation categories (global *p* = 0.004). The AUC was significantly higher in decompensated right-sided HF than in decompensated left-sided HF (0.841 vs. 0.603, *p* < 0.001), whereas no significant differences were observed between APE and decompensated left-sided HF (0.669 vs. 0.603, *p* = 0.670) or between cardiogenic shock and decompensated left-sided HF (0.485 vs. 0.603, *p* = 0.463). In contrast, discriminatory performance did not differ significantly between HFrEF and HFpEF (AUC 0.647 vs. 0.669, *p* = 0.784) or between patients without and with diabetes (AUC 0.629 vs. 0.679, *p* = 0.534).

### 3.4. Serial hs-cTnT Patterns and In-Hospital Mortality

A total of 334 patients had both baseline and repeated hs-cTnT measurements, including 35 patients who died during hospitalization. Compared with patients who had baseline hs-cTnT measurement only (*n* = 319), those undergoing repeated hs-cTnT assessment (*n* = 334) differed in several baseline characteristics (Table 5). Patients with repeated measurements had higher baseline hs-cTnT concentrations (62.5 ± 86.5 vs. 42.0 ± 46.1 pg/mL, *p* < 0.001) and higher urea levels (68.8 ± 43.4 vs. 60.9 ± 31.3 mg/dL, *p* = 0.007), as well as lower LVEF (42.7 ± 13.7% vs. 45.1 ± 14.6%, *p* = 0.029) and lower TAPSE (17.8 ± 4.7 vs. 19.0 ± 5.9 mm, *p* = 0.005). LVEF < 50% was more frequent among patients undergoing repeated hs-cTnT (62.6% vs. 50.8%, *p* = 0.002). In-hospital mortality was numerically higher in patients with repeated measurements (10.5% vs. 7.5%), although the difference was not statistically significant (*p* = 0.188).

**Figure 6:**
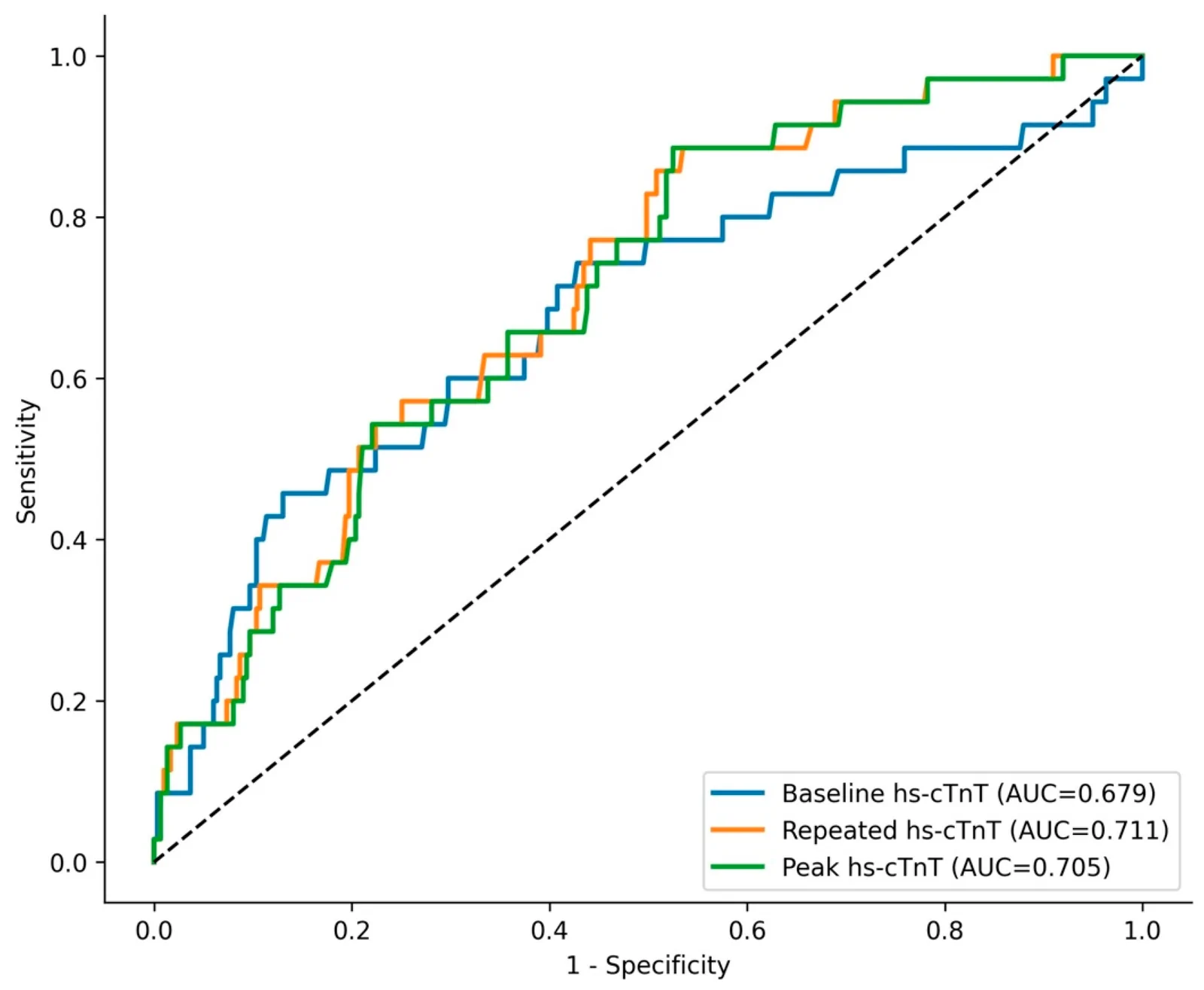
ROC curves for serial hs-cTnT measurements for in-hospital mortality. The dashed diagonal line represents the line of no discrimination.

Among the 334 patients with repeated hs-cTnT measurements, including 35 in-hospital deaths, baseline, repeated and peak hs-cTnT values were each associated with in-hospital mortality (Table 6). Repeated hs-cTnT showed the numerically highest discrimination (AUC 0.711, 95% CI 0.620–0.795, *p* < 0.001), followed by peak hs-cTnT (AUC 0.705, 95% CI 0.616–0.787, *p* < 0.001) and baseline hs-cTnT (AUC 0.679, 95% CI 0.570–0.782, *p* < 0.001) (Figure 6). Parameters reflecting relative hs-cTnT change showed limited prognostic performance. However, paired DeLong comparisons showed no significant difference between repeated and baseline hs-cTnT (ΔAUC 0.032, 95% CI −0.044 to 0.108, *p* = 0.413), between peak and baseline hs-cTnT (ΔAUC 0.026, 95% CI −0.048 to 0.099, *p* = 0.493), or between repeated and peak hs-cTnT (ΔAUC 0.006, 95% CI −0.004 to 0.016, *p* = 0.222). 

The incremental contribution of repeated hs-cTnT was further evaluated by comparing a baseline multivariable model with an extended model additionally incorporating repeated hs-cTnT. After adjustment for age, SBP, serum creatinine, NT-proBNP, hemoglobin and baseline hs-cTnT, each doubling of repeated hs-cTnT was associated with higher odds of in-hospital mortality (adjusted OR 1.59, 95% CI 1.14–2.21, *p* = 0.006). The addition of repeated hs-cTnT significantly improved the overall model fit (likelihood-ratio χ^2^ = 7.13, *p* = 0.008). The AUC increased from 0.750 in the baseline model to 0.777 in the extended serial model; however, this improvement in discrimination was not statistically significant (ΔAUC 0.026, DeLong *p* = 0.205). The Brier score decreased modestly from 0.0836 to 0.0816. Following bootstrap internal validation, optimism-corrected AUCs were 0.704 for the baseline model and 0.731 for the extended serial model, with corresponding optimism-corrected calibration slopes of 0.81 and 0.80.

**Figure 7:**
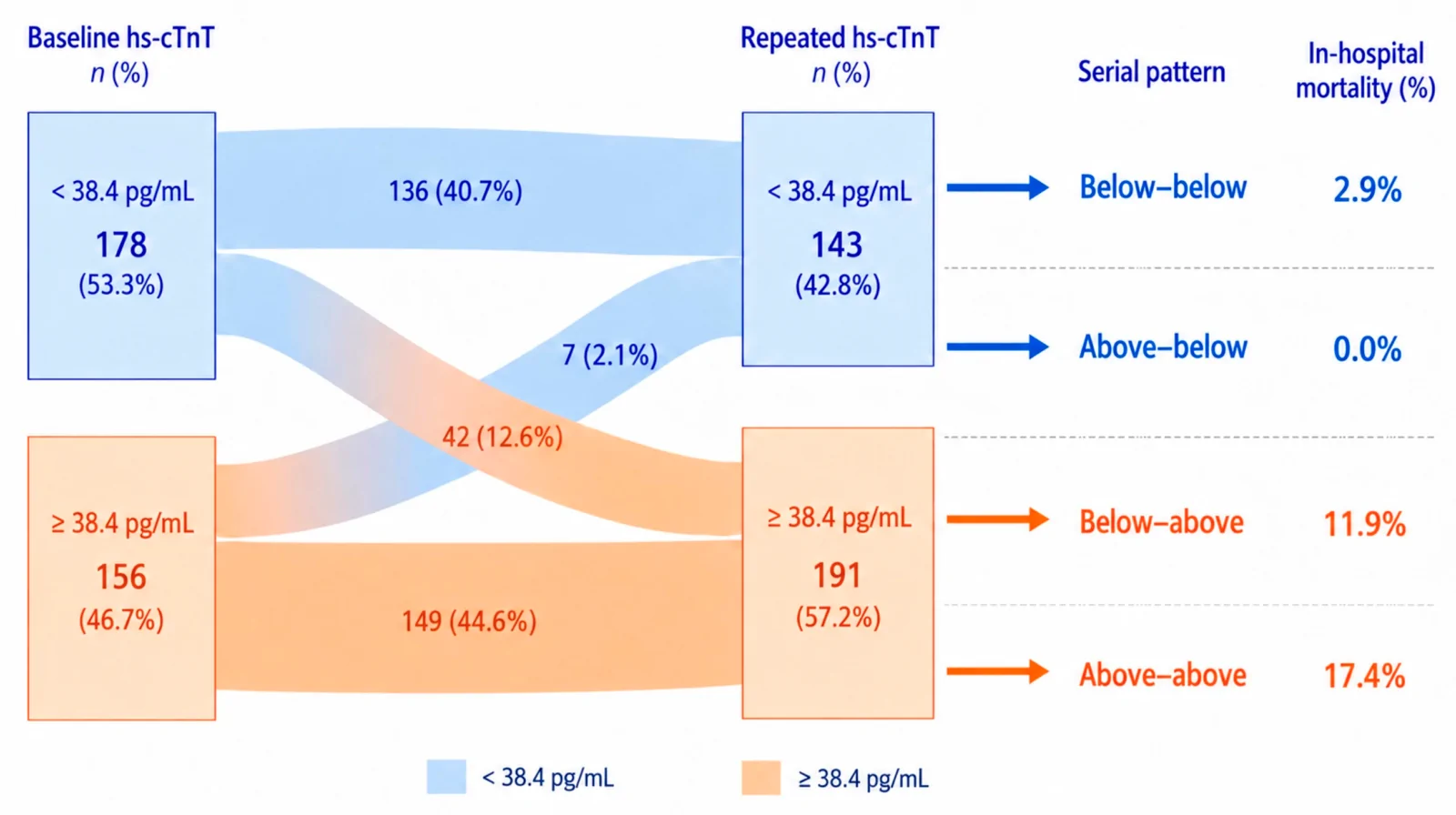
Serial hs-cTnT transition patterns according to threshold and associated in-hospital mortality.

Using the previously defined hs-cTnT threshold of 38.4 pg/mL, patients were further classified into four patterns: below-below, below-above, above-above and above-below. In-hospital mortality was 2.9% (4/136) in the below-below group, 11.9% (5/42) in the below-above group, 17.4% (26/149) in the above-above group and 0% (0/7) in the above-below group (Figure 7). The above-below subgroup was very small (*n* = 7), with no in-hospital deaths observed. The above-above hs-cTnT pattern was associated with significantly higher in-hospital mortality compared with all other serial patterns combined (OR 4.13, 95% CI 1.87–9.13, *p* < 0.001).

**Figure 8:**
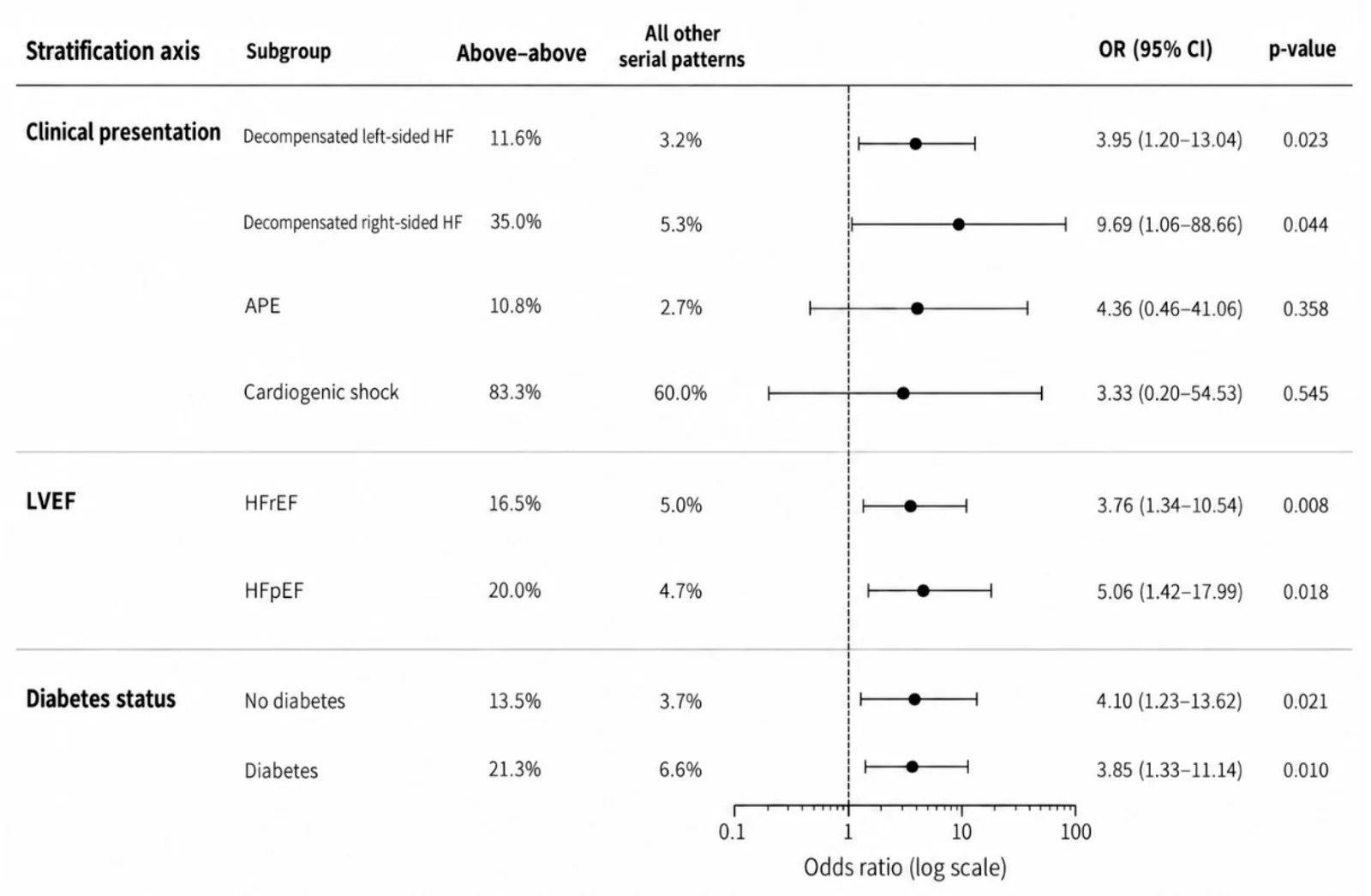
Association of the above-above hs-cTnT pattern with in-hospital mortality across exploratory subgroups.

The association between the above-above hs-cTnT pattern and in-hospital mortality was further evaluated according to clinical presentation, LVEF category, and diabetes status (Figure 8). Among patients with decompensated left-sided HF, in-hospital mortality was higher in the above-above group than among patients with all other serial patterns combined (11.6% vs. 3.2%; OR 3.95, 95% CI 1.20–13.04, *p* = 0.023). In patients with decompensated right-sided HF, mortality was 35% in the above-above group compared with 5.3% among those with other serial patterns (OR 9.69, 95% CI 1.06–88.66, *p* = 0.044). In patients with APE, mortality was numerically higher in the above-above group (10.8% vs. 2.7%; OR 4.36, 95% CI 0.46–41.06, *p* = 0.358). Among patients with cardiogenic shock, mortality remained high in both groups (83.3% vs. 60.0%; OR 3.33, 95% CI 0.20–54.53, *p* = 0.545). 

According to LVEF category, the above-above hs-cTnT pattern was associated with higher in-hospital mortality in both HFrEF and HFpEF. In HFrEF, mortality was 16.5% in the above-above group compared with 5.0% among patients with other serial patterns (OR 3.76, 95% CI 1.34–10.54, *p* = 0.008). In HFpEF, the corresponding mortality rates were 20.0% and 4.7%, respectively (OR 5.06, 95% CI 1.42–17.99, *p* = 0.018). 

When evaluated according to diabetes status, the above-above hs-cTnT pattern was associated with higher in-hospital mortality both in patients without diabetes (13.5% vs. 3.7%; OR 4.10, 95% CI 1.23–13.62, *p* = 0.021) and in patients with diabetes (21.3% vs. 6.6%; OR 3.85, 95% CI 1.33–11.14, *p* = 0.010). 

In a parsimonious bias-reduced Firth logistic regression model adjusted for age, SBP, serum creatinine, NT-proBNP and hemoglobin, the above-above hs-cTnT pattern remained associated with in-hospital mortality (adjusted OR 2.32, 95% profile penalized-likelihood CI 1.02–5.61, *p* = 0.045) (Table 7). Serum creatinine was also associated with in-hospital mortality (OR per doubling 1.88, 95% CI 1.06–3.38, *p* = 0.032). Sensitivity analyses incorporating TAPSE, RV-RA gradient, or urea in similarly constrained models yielded directionally consistent estimates for the above-above hs-cTnT pattern, although CIs widened in some models, consistent with the limited number of outcome events.

## 4. Discussion

In this single-center cohort of patients hospitalized with DHF, baseline hs-cTnT was associated with in-hospital mortality, although its standalone discriminatory performance was moderate. The internally-derived 38.4 pg/mL threshold identified patients at higher risk but showed limited stability on bootstrap validation. Formal interaction testing demonstrated heterogeneity according to clinical presentation, driven predominantly by a stronger association in decompensated right-sided than left-sided HF, whereas no evidence of effect modification was observed according to LVEF category or diabetes status. In the serial subgroup, repeated hs-cTnT remained associated with mortality after multivariable adjustment but did not significantly improve discrimination, and threshold-based transition patterns were exploratory. These findings support hs-cTnT as a complementary component of integrated risk assessment rather than as a standalone or treatment-directing biomarker.

### 4.1. High-Sensitivity Troponin as a Marker of Myocardial Injury and Short-Term Risk in DHF

Cardiac troponin elevation is common in patients hospitalized with DHF and may reflect myocardial injury related to increased wall stress, impaired perfusion, tachyarrhythmia, hypoxemia, renal dysfunction and other mechanisms occurring in the absence of acute myocardial ischemia [18,19,21,22,23]. In the present cohort, baseline hs-cTnT was associated with in-hospital mortality both when evaluated continuously and according to the study-derived threshold. However, its overall discriminatory performance was only moderate (AUC 0.658), indicating that a single admission measurement captures only part of the short-term risk profile of patients with DHF. Accordingly, hs-cTnT should be considered a component of integrated risk assessment rather than a standalone prognostic classifier.

The internally derived threshold of 38.4 pg/mL identified patients at higher in-hospital risk, but its variability on bootstrap validation supports an exploratory interpretation and the need for external validation before clinical application. This prognostic threshold should also be distinguished from the assay-specific 99th-percentile upper reference limit, which identifies myocardial injury rather than mortality risk. In addition, the limited incremental contribution of baseline hs-cTnT beyond NT-proBNP in the present cohort supports a multimarker rather than a single-biomarker approach to risk assessment. Emerging multimodal strategies integrating natriuretic peptide information with routinely available clinical signals, including AI-supported ECG approaches, further reinforce this concept [33,34]. 

### 4.2. Baseline hs-cTnT Across Clinical Presentation, LVEF Category and Diabetes Status

Clinical presentation emerged as the stratification axis showing the clearest heterogeneity in the prognostic association of baseline hs-cTnT. Using the contemporary 2026 ESC framework [1], formal interaction testing demonstrated significant effect modification by clinical presentation, driven predominantly by a stronger association between increasing hs-cTnT and in-hospital mortality in decompensated right-sided HF compared with decompensated left-sided HF. Formal AUC comparisons were consistent with this finding, with significantly greater discrimination in the right-sided than in the left-sided decompensated HF. By contrast, estimates in APE and cardiogenic shock were less precise, and the limited number of events in these subgroups precludes interpreting non-significant findings as evidence of absent prognostic association. These results support the concept that prognostic meaning of myocardial injury may vary according to the clinical and hemodynamic context of HF decompensation [2,6]. 

In contrast, the present data did not demonstrate significant effect modification according to LVEF. Baseline hs-cTnT was associated with mortality in both HFrEF and HFpEF, but neither the formal interaction test nor the direct comparison of AUCs supported a significant difference between the two contemporary LVEF categories. Previous studies have reported differences in the prognostic performance of hs-cTnT across the LVEF spectrum, but we found no statistical evidence that LVEF category modified the association between hs-cTnT and in-hospital mortality [35]. An exploratory analysis within HFpEF also examined patients with LVEF 50–64% and those with supra-normal LVEF ≥65%. Although emerging evidence suggests that supra-normal LVEF may represent a biologically and prognostically distinct HF phenotype [36,37], no significant hs-cTnT-by-LVEF subgroup interaction was observed in our cohort. Given that only three deaths occurred among patients with LVEF ≥65%, this finding should be considered exploratory and insufficient to exclude clinically relevant heterogeneity.

Similarly, diabetes status did not significantly modify the association between baseline hs-cTnT and mortality, despite higher absolute mortality among patients with diabetes and hs-cTnT above the study-derived threshold. Diabetes should therefore be regarded here as a clinically relevant comorbidity rather than as evidence of a distinct hs-cTnT prognostic phenotype. Underlying HF etiology may represent an additional source of heterogeneity: ischemic etiology was more frequent among non-survivors in the present cohort, while non-ischemic etiologies encompass diverse myocardial substrates that may influence both troponin release and clinical trajectory [38]. Because specific non-ischemic etiologies were not characterized with sufficient granularity for robust subgroup analysis, this dimension could not be explored further. Prior studies have already established the prognostic relevance of hs-cTnT across LVEF categories and of serial hs-cTnT assessment [22,35]. Therefore, the principal contribution of the present study lies in integrating clinical presentation, contemporary LVEF classification, diabetes status and longitudinal hs-cTnT behavior within a single prospective DHF cohort.

### 4.3. Serial hs-cTnT Measurements and In-Hospital Mortality

Serial hs-cTnT measurements were associated with in-hospital outcome, but their incremental prognostic contribution should be interpreted cautiously. Repeated and peak hs-cTnT showed numerically higher discrimination than baseline hs-cTnT. However, paired DeLong comparisons demonstrated no statistically significant differences between their AUCs. When repeated hs-cTnT was added to a parsimonious baseline model including age, SBP, serum creatinine, NT-proBNP, hemoglobin and baseline hs-cTnT, the repeated measurement remained associated with mortality and improved overall model fit, although the increase in discrimination was modest and was not statistically significant. These findings are consistent with previous evidence on serial hs-cTnT in hospitalized HF, but do not establish that routine serial measurement improves clinical risk classification [22,23].

Using the internally derived 38.4 pg/mL threshold, the above-above pattern was associated with the highest in-hospital mortality. This terminology reflects persistence above a study-specific prognostic threshold rather than a biochemical elevation relative to the assay-specific URL. In the bias-reduced Firth model, the above-above pattern remained associated with mortality after adjustment for age, SBP, serum creatinine, NT-proBNP and hemoglobin, although the limited number of events warrants cautious interpretation.

The remaining transition patterns should be interpreted descriptively. In particular, only 7 patients followed an above-below trajectory and no deaths occurred in this subgroup. Therefore, these data do not support an interpretation that crossing below the study-derived threshold represents biological recovery or a prognostically protected state. Serial-pattern subgroup analyses according to clinical presentation, LVEF and diabetes status should likewise be regarded as exploratory.

### 4.4. Clinical Implications

Within the contemporary ESC framework, hs-cTnT should be interpreted as a complementary component of integrated clinical assessment rather than as a treatment-directing biomarker. Baseline and serial hs-cTnT measurements may contribute to short-term risk characterization when considered alongside clinical presentation, congestion and perfusion status, renal function, natriuretic peptides and echocardiographic findings. However, the observational design of the present study does not establish that hs-cTnT-guided monitoring or therapeutic escalation improves clinical outcomes. Management should therefore remain centered on identification and treatment of precipitating factors, appropriate decongestion, hemodynamic management according to congestion and perfusion status, and optimization of guideline-directed HF therapy [1]. Accordingly, the present findings support further investigation of hs-cTnT as an adjunct to integrated risk assessment rather than as a marker that should independently dictate clinical management.

### 4.5. Strengths and Limitations

The principal strength of this study is the prospective evaluation of baseline and serial hs-cTnT in a relatively large cohort of patients hospitalized with DHF, integrating contemporary clinical presentation and LVEF classifications with diabetes status and longitudinal biomarker assessment. The analyses incorporated formal interaction testing, direct comparisons of discriminatory performance, internally validated multivariable models and sensitivity analyses that reduce reliance on isolated subgroup-specific *p*-values. Nevertheless, the single-center design may limit generalizability, and the internally-derived 38.4 pg/mL prognostic threshold requires external validation before clinical application. Several subgroup analyses involving cardiogenic shock, supra-normal LVEF, and individual serial transition patterns included few outcome events and should therefore be considered exploratory. Moreover, multiple subgroup comparisons were performed without formal adjustment for multiplicity, increasing the possibility of chance findings; interpretation should consequently emphasize effect estimates, CI and interaction tests rather than isolated statistical significance. 

Serial hs-cTnT analyses are subject to additional limitations. Repeated measurements were available in only a subset of the cohort, and patients undergoing serial assessment differed in several baseline characteristics from those with admission measurements only. Repeat sampling was clinician-driven rather than protocolized, specific indications for repeat testing were not systematically recorded, and sampling intervals varied. Therefore, selection, indication and timing bias cannot be excluded. In addition, patients had to remain alive and hospitalized long enough to undergo repeat measurement, introducing potential survivor/landmark bias. Finally, although echocardiographic examinations were performed by experienced cardiologists before subsequent in-hospital outcome was known, formal interobserver and intraobserver reproducibility analyses were not performed. These limitations should be considered when interpreting the phenotype-stratified and longitudinal hs-cTnT findings. Detailed information on treatment intensity, dosing, timing, vasopressor-specific therapy and mechanical circulatory support was not systematically captured with sufficient granularity for treatment-adjusted analyses. Therefore, potential treatment-related effects on serial hs-cTnT trajectories and outcomes could not be assessed. 

## 5. Conclusions

Baseline hs-cTnT was associated with in-hospital mortality in patients hospitalized with DHF, although its overall discriminatory performance was moderate. Clinical presentation significantly modified this association, driven predominantly by a stronger association in decompensated right-sided HF, whereas no significant modification effect was observed according to LVEF category or diabetes status. Repeated hs-cTnT remained associated with in-hospital mortality after multivariable adjustment but did not significantly improve discrimination. Threshold-based serial patterns, including the above-above trajectory, should be considered exploratory. Overall, hs-cTnT may complement integrated risk assessment in DHF, while external validation of the study-derived threshold and subgroup findings is required before clinical application. 

## Figures and Tables

**Table 1:** Baseline characteristics of patients with DHF according to in-hospital outcome.

Variable	Patients (*n* = 653)	Survivors (*n*= 594)	Non-Survivors (*n* = 59)	*p*-Value
Age, years	74.4 ± 11.8	74.0 ± 11.9	77.7 ± 9.5	0.007
Male sex, %	49.5	49.3	50.8	0.823
Hypertension, %	92.5	93.1	86.4	0.071
Diabetes mellitus, %	43.0	42.1	52.5	0.122
Dyslipidemia, %	87.4	88.9	72.9	<0.001
Obesity, %	44.2	44.8	37.9	0.316
Smoking, %	25.5	25.5	25.4	0.983
Atrial fibrillation/Atrial flutter, %	62.6	62.0	69.5	0.254
Decompensated left-sided HF, %	66.9	68.9	47.5	<0.001
Decompensated right-sided HF, %	11.0	9.8	23.7	0.001
APE, %	19.4	20.4	10.2	0.059
Cardiogenic shock, %	2.6	1.0	18.6	<0.001
Ischemic etiology, %	50.8%	49.0%	69.5%	0.003
SBP, mmHg	146.7 ± 32.0	147.8 ± 31.9	135.7 ± 31.1	0.006
Heart rate, bpm	98.6 ± 29.8	98.3 ± 29.8	101.9 ± 29.3	0.372
Oxygen saturation, %	93.2 ± 5.5	93.3 ± 5.5	92.4 ± 4.9	0.192
NT-proBNP, pg/mL	9476.1 ± 12,403.0	8715.3 ± 12,024.1	17,136.2 ± 13,619.7	<0.001
hs-cTnT, pg/mL	52.5 ± 70.5	48.5 ± 60.9	92.2 ± 126.9	0.011
Lactate, mmol/L	2.2 ± 1.5	2.2 ± 1.3	3.0 ± 2.7	0.052
Sodium, mmol/L	138.3 ± 5.4	138.4 ± 5.3	137.0 ± 6.0	0.125
Creatinine, mg/dL	1.41 ± 0.81	1.37 ± 0.78	1.78 ± 0.94	0.002
Urea, mg/dL	68.3 ± 39.6	65.0 ± 36.3	97.4 ± 53.5	<0.001
Hemoglobin, g/dL	12.5 ± 2.5	12.6 ± 2.4	11.3 ± 2.4	0.002
CRP, mg/L	25.6 ± 45.0	23.9 ± 42.7	41.0 ± 60.9	0.064
LVEF, %	43.8 ± 14.2	44.2 ± 13.9	40.3 ± 16.6	0.089
HFrEF, LVEF < 50%	56.8	56.2	62.7	0.338
HFpEF, LVEF ≥ 50%	43.2	43.8	37.3	0.338
TAPSE, mm	18.4 ± 5.4	18.5 ± 5.3	16.6 ± 5.5	0.012
RV-RA gradient, mmHg	31.9 ± 13.0	31.5 ± 12.8	36.3 ± 14.4	0.016
IVC diameter, mm	17.1 ± 5.5	17.0 ± 5.4	18.5 ± 5.7	0.057

APE, acute pulmonary edema; SBP, systolic blood pressure; NT-proBNP, N-terminal pro-B-type natriuretic peptide; hs-cTnT, high-sensitivity cardiac troponin T; CRP, C-reactive protein; LVEF, left ventricular ejection fraction; HF, heart failure; HFrEF, heart failure with reduced ejection fraction; HFpEF, heart failure with preserved ejection fraction; TAPSE, tricuspid annular plane systolic excursion; RV-RA gradient, right ventricle-right atrium gradient; IVC diameter, inferior vena cava diameter.

**Table 2:** Prognostic performance of baseline hs-cTnT according to clinical presentation.

Clinical Presentation	*n*	In-Hospital Mortality, *n* (%)	AUC	95% CI	*p*-Value
Decompensated left-sided HF	437	28 (6.4%)	0.603	0.488–0.717	0.069
Decompensated right-sided HF	72	14 (19.4%)	0.841	0.705–0.976	<0.001
APE	127	6 (4.7%)	0.669	0.427–0.912	0.164
Cardiogenic shock	17	11 (64.7%)	0.485	0.189–0.781	0.960

APE, acute pulmonary edema; AUC, area under the curve; CI, confidence interval, HF, heart failure.

**Table 3:** Prognostic performance of baseline hs-cTnT according to LVEF category.

LVEF Category	*n*	In-Hospital Mortality, *n* (%)	AUC	95% CI	*p*-Value
HFrEF	371	37 (10.0%)	0.647	0.547–0.747	0.003
HFpEF	282	22 (7.8%)	0.669	0.541–0.797	0.008

AUC, area under the curve; CI, confidence interval; HFrEF, heart failure with reduced ejection fraction; HFpEF, heart failure with preserved ejection fraction; LVEF, left ventricular ejection fraction.

**Table 4:** Prognostic performance of baseline hs-cTnT according to diabetes status.

Diabetes Status	*n*	In-Hospital Mortality, *n* (%)	AUC	95% CI	*p*-Value
No diabetes	372	28 (7.5%)	0.629	0.504–0.739	0.023
Diabetes	281	31 (11.0%)	0.679	0.576–0.784	0.001

AUC, area under the curve; CI, confidence interval.

**Table 5:** Baseline characteristics of patients according to availability of repeated hs-cTnT measurements.

Variable	Repeated hs-cTnT (*n* = 334)	Baseline hs-cTnT Only (*n* = 319)	*p*-Value
Age, years	75.2 ± 10.6	73.5 ± 12.8	0.073
Male sex, %	49.8	49.1	0.840
Hypertension, %	92.5	92.5	0.985
Diabetes status, %	45.2	40.8	0.250
Dyslipidemia, %	86.5	88.4	0.470
Obesity, %	44.9	43.4	0.697
Smoking, %	27.5	23.4	0.228
Atrial fibrillation/flutter, %	62.9	62.4	0.897
Ischemic etiology, %	58.1	43.3	<0.001
Decompensated left-sided HF, %	62.9	71.2	0.024
Decompensated right-sided HF, %	11.7	10.3	0.587
APE, %	22.2	16.6	0.074
Cardiogenic shock, %	3.3	1.9	0.257
SBP, mmHg	149.4 ± 32.6	143.8 ± 31.0	0.024
Heart rate, bpm	98.0 ± 29.1	99.3 ± 30.5	0.593
Oxygen saturation, %	92.9 ± 5.9	93.5 ± 5.0	0.182
NT-proBNP, pg/mL	9921.2 ± 12,296.4	9010.2 ± 12,516.0	0.349
Baseline hs-cTnT, pg/mL	62.5 ± 86.5	42.0 ± 46.1	<0.001
Lactate, mmol/L	2.2 ± 1.4	2.1 ± 1.4	0.163
Sodium, mmol/L	138.6 ± 5.6	138.6 ± 4.5	0.990
Creatinine, mg/dL	1.43 ± 0.80	1.39 ± 0.81	0.447
Urea, mg/dL	68.8 ± 43.4	60.9 ± 31.3	0.007
Hemoglobin, g/dL	12.7 ± 2.2	12.5 ± 2.5	0.374
CRP, mg/L	25.2 ± 40.8	21.8 ± 40.8	0.285
LVEF, %	42.7 ± 13.7	45.1 ± 14.6	0.029
HFrEF, LVEF < 50%	62.6	50.8	0.002
HFpEF, LVEF ≥ 50%	37.4	49.2	0.002
TAPSE, mm	17.8 ± 4.7	19.0 ± 5.9	0.005
RV-RA gradient, mmHg	31.4 ± 10.8	32.5 ± 14.9	0.295
IVC diameter, mm	17.4 ± 5.7	16.9 ± 5.3	0.220
In-hospital mortality, %	10.5	7.5	0.188

APE, acute pulmonary edema; SBP, systolic blood pressure; NT-proBNP, N-terminal prohormone of brain natriuretic peptide; hs-cTnT, high-sensitivity cardiac troponin T; CRP, C-reactive protein; LVEF, left ventricular ejection fraction; HFrEF, heart failure with reduced ejection fraction; HFpEF, heart failure with preserved ejection fraction; TAPSE, tricuspid annular plane systolic excursion; RV-RA gradient, right ventricle-right atrium gradient; IVC diameter, inferior vena cava diameter.

**Table 6:** Prognostic performance of serial hs-cTnT parameters for in-hospital mortality.

Serial hs-cTnT	AUC	95% CI	*p*-Value
Baseline hs-cTnT	0.679	0.570–0.782	<0.001
Repeated hs-cTnT	0.711	0.620–0.795	<0.001
Peak hs-cTnT	0.705	0.616–0.787	<0.001
Absolute delta magnitude	0.613	0.501–0.717	0.029
Relative delta, %	0.528	0.409–0.647	0.590

AUC, area under the curve; CI, confidence interval; hs-cTnT, high-sensitivity cardiac troponin T.

**Table 7:** Multivariable Firth logistic regression analysis for in-hospital mortality in patients with serial hs-cTnT measurements.

Variable	Adjusted OR	95% CI	*p*-Value
Above-above hs-cTnT pattern	2.32	1.02–5.61	0.045
Age, per 10-year increase	1.08	0.74–1.58	0.700
SBP, per 10-mmHg increase	0.92	0.82–1.03	0.142
Serum creatinine, per doubling	1.88	1.06–3.38	0.032
NT-proBNP, per doubling	1.19	0.92–1.54	0.183
Hemoglobin, per 1-g/dL increase	0.93	0.79–1.09	0.378

SBP, systolic blood pressure; NT-proBNP, N-terminal pro-B-type natriuretic peptide; hs-cTnT, high-sensitivity cardiac troponin T.

## Data Availability

The data used in this study are available from the corresponding author upon request. The data are not publicly accessible due to patient privacy and confidentiality constraints.

## Abbreviations

The following abbreviations are used in this manuscript:

ACS	Acute Coronary Syndrome
APE	Acute Pulmonary Edema
AUC	Area Under the Curve
CRP	C-reactive Protein
CI	Confidence Interval
DHF	Decompensated Heart Failure
ED	Emergency Department
HF	Heart Failure
HFpEF	Heart Failure with preserved Ejection Fraction
HFrEF	Heart Failure with reduced Ejection Fraction
hs-cTnT	High-sensitivity cardiac Troponin T
IQR	Interquartile range
IVC	Inferior Vena Cava
LVEF	Left Ventricular Ejection Fraction
NT-proBNP	N-terminal pro-B-type Natriuretic Peptide
OR	Odds ratio
RV-RA	Right Ventricular-Right Atrial
ROC	Receiver Operating Characteristic
SBP	Systolic Blood Pressure
SPSS	Statistical Package for Social Sciences
TAPSE	Tricuspid Annular Plane Systolic Excursion
URL	Upper Reference Limit
